# Towards a unified carbon accounting landscape

**DOI:** 10.1098/rsta.2023.0260

**Published:** 2024-09-23

**Authors:** Lewis J. McDonald, Jose Luis Hernandez Galvan, Chukwudi Emelue, Ariane S. S. Pinto, Neha Mehta, Taofeeq Ibn-Mohammed, Thomas Fender, Jonathan Radcliffe, Alok Choudhary, Marcelle C. McManus

**Affiliations:** ^1^ Institute for Sustainability, University of Bath, Bath BA2 7AY, UK; ^2^ Birmingham Energy Institute, University of Birmingham, Birmingham B15 2TT, UK; ^3^ WMG, University of Warwick, Coventry CV4 7AL, UK

**Keywords:** carbon, climate-justice, circular-economy, global-emissions, life cycle

## Abstract

The overarching purpose of carbon accounting is to reduce carbon emissions to meet net-zero targets and minimize the impact of climate change. However, the plethora of methods and approaches used means that products and systems sometimes cannot easily be compared. The mix of regional and life cycle-based systems can mean that we lack global oversight of our emissions and impact. In some situations where a regional approach is used, industry/business/regions are incentivized to reduce their own/territorial emissions, which can mean that an optimal global solution is not adopted. Countries where grid emissions are higher can be selected for production because it reduces regional (not global) carbon levels. Furthermore, these can be areas where the climate impact may be felt the most: not the just transition we aspire to. Our work provides an analysis of the current system together with its challenges and limitations, paving the way towards a more unified framework to create climate justice together with transparent and comparable accounting methodology for industry and regions alike.

This article is part of the discussion meeting issue ‘Green carbon for the chemical industry of the future’.

## 1. Introduction

The ability to measure and account for carbon consistently and transparently is critical to effective decarbonization. However, the current carbon accounting space is filled with numerous standards: Greenhouse Gas protocol (GHG protocol), ISO & BSI and Science-Based Targets initiative (SBTi) approaches characterized by varying methodologies, tools and variation in flexibility, which leads to inconsistency as the complexities of the system increase [1]. Pulls in different directions in carbon reporting and disclosure mean extra cost to companies, variability and ultimately a lack of ability to compare products and systems and their impact on climate change. In addition, the quality of the assessment is varied, and as the complexity of the system increases, the inconsistency in approaches has made this variability and lack of comparability more problematic [2]. Local industries in particular will suffer the consequences if methodologies do not recognize their contribution to a circular economy and how they can integrate carbon accounting into normal business practices. The lack of an appropriate accounting method means that carbon is not factored into business planning cycles; hence, there is a lack of investment in tech/processes that could reduce emissions.

To find appropriate systems for carbon accounting, several critical issues first need to be addressed: the complexity of global supply chains and how sub-components of this are measured and accounted for consistently across the piece; the difference between product and regional based accounting approaches; and the additional complexity of measuring and attributing credits and burdens in a more circular economy. Carbon accounting suffers from having two main purposes: incentivizing actors to do the right thing and measuring carbon for reporting and meeting targets.

These issues can be illustrated in the case of industrial clusters, which have developed in some regions and countries to help meet net-zero targets and to enable and encourage innovation and collaboration between industries. They have clear targets and needs, and businesses within them are encouraged to work together to reduce emissions. In some cases, this means that companies are looking at a more circular local economy; for example, one company using another’s ‘waste’ as a resource. Intuitively, this would mostly be considered an environmental benefit, but it can come with logistical difficulties, for example, how to determine who would get any carbon or environmental credit when one company uses another’s ‘waste’ to make a product. Within life cycle assessment (LCA) ISO standards, there are several options for burden/credit sharing for recycling and using recycled products, but this can be inconsistently applied and is not carried over into carbon standards.

This gets more complex when emission mitigation/offsetting—e.g. carbon capture, utilization and storage (CCUS)—is considered. The products could lock carbon for different time frames, influencing the emissions over time drastically compared with processes or systems other than CCUS. Although dynamic characterization factors can be used, here the main temporal aspect to be evaluated is how long the carbon is captured as a product or within a longer-term storage system and how to minimize emissions along the supply chain (process efficiency). Work on nature-based carbon storage (tonne/yr) has been proposed [3], which could be incorporated into accounting frameworks. Steininger *et al*. [4] note that for a system capturing and storing carbon, uncertainty in the accounting will surround the process. This becomes more complex when the carbon is captured and used by a neighbouring company to create a circular carbon economy. Temporal aspects could be decisive in assessing the reduction of the emissions from each cluster.

Although significant CCUS systems are not considered as widespread in the UK or globally, they are in evidence already through recycled plastics and similar materials. More complex CCUS systems are commonly considered critical to our pathway to net zero, and so as we move towards a circular economy where carbon is captured in materials over several life cycles, understanding and mapping this impact to the global environment as well as to the local businesses and industry is crucial to both decarbonization and the viability of local industries.

The critical areas of carbon storage, geography and data drive accounting methods selected, along with sectoral (e.g. energy and electricity [5]) and regulatory requirements. The requirement for a framework for carbon accounting in the digital sector has been highlighted [6]. Further fragmentation, however, will not solve the issues associated with the use of inconsistent methods, and therefore a more overarching framework approach is required.

## 2. Current drivers and standards for carbon accounting

Major standards such as GHG protocol, ISO (BSI), PAS 2050, etc., provide some level of standardized basis for corporate carbon accounting and reporting that are already being implemented by countries and businesses. In the UK, policy mechanisms such as the Emission Trading Scheme (ETS) and Streamlined Energy and Carbon Reporting (SECR) system are influencing businesses’ carbon accounting practices [7]. There are also disclosure mechanisms such as the Carbon Disclosure Project (CDP), SBTi, Energy Savings Opportunity Scheme (ESOS), the Climate Change Agreement (CCA) and the International Sustainability Standard Board (ISSB). ISSB builds on the work of the Taskforce on Climate-Related Financial Disclosure (TCFD), Climate Disclosure Standard Board (CDSB), Value Reporting Foundation’s Integrated Reporting Framework, World Economic Forum’s Stakeholder Capitalism Metrics, as well as industry-based Sustainability Accounting Standard Board (SASB). This section reviews the accounting methods that can be applied at an aggregated macro level and for individual products or organizations.

### (a) Macro-level accounting methods

Carbon accounting methods are often selected by the need to attach the responsibility for the emissions to a particular actor within the supply chain of the good or service: raw material extractor, producer, consumer or income receptor. However, the adoption of these methods lacks consistency with the current standards/frameworks and policies as is highlighted in table 1, which maps these.

**Table 1.** Methodological approach adopted in current regulations, standards and framework.

	territorial based	production based	consumption based	income based	EEMRIO	ETS	CBAM
GHG protocol	✓	✓	—	—	—	—	—
ISO 14067	—	✓	✓	—	✓	—	—
PAS 2050	✓	✓	✓	—	—	—	—
compliance market-based mechanism—ETS	✓	—	✓	—	—	✓	✓
SECR	—	✓	✓	—	—	—	—
CDP	—	✓	—	—	—	—	—
SBTi	—	—	—	—	—	—	—
ESOS	✓	—	✓	—	—	—	—
CCA	✓	—	—	—	—	—	—
ISSB	—	—	—	—	—	—	—
TCFD	—	—	—	—	—	—	—
CDSB	—	—	—	—	—	—	—
value reporting foundation’s integrated reporting framework	—	—	—	—	—	—	—
World Economic Forum’s stakeholder capitalism metrics	—	—	—	—	—	—	—
SASB	—	✓	—	—	—	—	—

#### (i) Production-based accounting

Production-based accounting was introduced globally in the Kyoto Protocol, and countries that ratified the Paris Agreement are required to report their emissions using this method [8]. The objective of the production-based approach is to identify main pollution emitters within the supply chain prompting them to reduce emissions by using renewable energy and cleaner technologies, implementing energy efficiency and optimization strategies or carbon capture utilization and storage.

A production-based accounting approach follows the ‘polluter pays’ principle, where material extraction and associated emissions are allocated to the actor who drives the economic activity, aiming to identify the most important direct emitter of such activity [9]. The calculation is made within a given regional boundary; therefore, consumption of goods exported from outside of the region under study is not considered. This approach can lead countries or companies to offshore carbon-intensive industries to have lower impacts at a local level. This incentivizes the importation of products and a focus on adding value through less polluting local activities. However, as the countries that provide the offshore services may have larger production impacts, this regionalized approach does not always provide optimal carbon reduction activities at a global level.

#### (ii) Consumption-based accounting

Consumption-based accounting was developed to address the shortcomings of production-based, namely the lack of international trade. It assigns burdens from global supply chains to the end user of products and services, so the final or intermediate consumer can identify any impact within the supply chain of the goods.

Accounting for the emissions at the point of consumption allows analysts to split burdens from international trade between trade partners, but also for production and distribution emissions [10]. As a result of this, consumers can help suppliers improve their environmental performance or can replace them with lower impact products and services that offer the same function, nevertheless, replacing inputs does not have an impact if the production method or the product design is modified [11].

Actors that are net importers will have higher consumption-based emissions than production-based emissions and vice versa for net exporters. Karakaya *et al*. [8] attest that consumption-based accounting is the more appropriate method as it avoids import and export emissions, rendering transparency on the environmental impact of goods, a crucial aspect of this approach.

Contrary to production-based methods where offshoring would be a disadvantage for some undeveloped countries in terms of emissions attributed to them, the consumption-based approach aims to cede burdens for emissions associated with exported production and accepts responsibility for the embodied emissions of the imported goods and services. This can drive the implementation of mitigation measures in offshore countries while promoting the reduction of consumption levels at the local scale [10].

With this ‘shared’ responsibility, the consumption-based approach seeks to have a global impact due to the distribution of carbon emissions among countries to benefit the development of international climate change policies. Nevertheless, effectiveness due to political issues and methodological complexities for different geographies is a limitation for its wider application [12].

#### (iii) Income-based accounting

Income-based accounting allocates the responsibility for carbon emissions to global economic actors based on the benefits that they gain related to their activities and the emissions that they enable in their downstream supply chains. It was developed as an alternative to consumption-based accounting to assign burdens to those who financially benefit rather than consumers [13]. Thus, all emissions produced along a supply chain are attributed to specific actors and countries according to the added value that each of them contributes, which is directly related to the income they earn. Final emissions may also be attributed to the last stage of the supply chain, which is the consumer or final user, in the function of the country of residence.

#### (iv) Environmentally extended multi-regional input–output models

Environmentally extended multi-regional input–output (EEMRIO) models link standard economic matrices of different countries with natural resources and pollution accounts, as a way of tracking the use of natural resources as non-monetary inputs for a supply-chain process [14]. This method relies on regional material, energy and economic data into and out of a given region [15] to quantify and assign responsibilities within the economic activity for changes in natural resource use and pollution levels. It is included in this list as it is a common approach to applying consumption- and income-based accounting (e.g. [11,16–18]).

A top–down LCA modelling framework based on the EEMRIO analytical framework is developed from the general input–output (IO) model and used as the basis to analyse the global supply-chain environmental sustainability and the resultant implications for the UK basic metal sector in comparison with top global metal-producing countries. The basic IO framework, based on the seminal work of Leontief [19], models the production and consumption structure of an economy using records of monetary transactions representing the flows of resources from the products and services sectors to the industry-producing sectors. The Environmentally Extended Input–Output (EEIO)’s underlying assumption is that products and services produced in an economy expressed in monetary form are transformed into physical flows [20]. This allows the capturing of the economy-wide physical products and services requirements used by intermediate industries in the economy. By linking financial transaction data with environmental discharge data (e.g. GHG emissions, material use, toxicity and eutrophication), the environmental implications of global production and consumption activities can be evaluated [21–23].

The EEMRIO integrated IO tables from multiple countries into a consistent and unified multi-region framework [9]. This allows the environmental emission intensities of production technology, the supply-chain requirements alongside the inter- and intra-trade relationships among different countries captured in the model, to be compared. It also supports the analysis of different scenarios (e.g. whether there is evidence for carbon leakage or not) and the impact that technological and supply-chain changes in one country would have on other countries.

This method enables the utilization of monetary IO factors and different environmental factors as required by different sectors of an economy. It also allows allocation of global domestic extraction according to the country of the final user. However, EEMRIO does not specify how the changes take place spatially at a local level within a country or area in trade partner countries [24].

#### (v) ETS and carbon border adjustment mechanisms

ETS, sometimes referred to as cap-and-trade mechanisms, set limits on sectoral emissions, which decline over time. Businesses are provided with (or buy) emissions allowances annually in terms of tCO_2_; if they emit more than their allowances, they must buy a quantity through a market (or face penalties); if they emit less, they can sell their allowances on the market (or hold on to them). This market trading introduces a price on carbon emissions.

The UK was originally part of the European Union (EU) ETS but has been maintaining its own scheme since 2021, following its departure from the EU in 2020. The UK ETS is not an all-encompassing scheme; many sectors such as energy from wastes and non-pipeline carbon capture are not included [7]. Furthermore, some sectors such as aviation receive preferential treatment in the form of free allocation of allowances.

The EU’s Carbon Border Adjustment Mechanism (CBAM) has been developed to charge a levy on imported goods whose emissions have not been priced (or under-priced compared with the EU) in their country of origin. Its purpose is to replace exemptions given to industries that have received free allowances in the past while reducing carbon leakage by ensuring that the carbon price of imports is equivalent to those of EU domestic production [25]. The CBAM came into effect on 1 October 2023 in a transitional phase covering six high-impact industries: cement, iron and steel, aluminium, fertilizers, electricity and hydrogen. The permanent system takes effect on 1 January 2026 and is intended to cover more than 50% of the emissions in the ETS; by 2030, all ETS goods are expected to be covered. Free allocation under the EU ETS will be phased out as the permanent CBAM is phased in the European Commission [26].

### (b) Product and organization-level carbon accounting

#### (i) LCA

LCA is a tool for assessing the life cycle impacts of products and processes. The ISO standards 14040 [27] and 14044 [28] describe best practices of LCA, divided into four steps: goal & scope definition, inventory analysis, impact assessment and interpretation. LCA can be used as a form of carbon accounting by using metrics such as global warming potential, where the carbon dioxide and equivalents associated with a product or process can be quantified.

Environmental product declarations (EPD), which is defined by ISO 14025 [29], for the quantification of environmental information is based on LCA and ISO 14040. EPD are growing in popularity as a means of improving the sustainability goals of organizations and providing consumers with accurate emission data. In Europe, EN 15804 [30] is being rolled out across the construction sector to quantify carbon associated with construction and improve the disclosure of emissions of specific products.

#### (ii) Scope 1, 2 and 3 emissions

As a measure towards limiting the global temperature to well below 2°C, the World Resource Institute developed the scope 1, 2 and 3 systems in the GHG protocol. The GHG protocol [31] defines the following three types of emissions at the organization level:

— Scope 1 emissions are direct emissions from operations that are owned or controlled by the reporting company; this could be running machinery to make products and combusting fuel onsite to power buildings and computers.
— Scope 2 emissions are indirect emissions created by the production of the energy that an organization buys. They are indirect emissions associated with the generation of consumed electricity, steam, heating or cooling an organization purchased or acquired.
— Scope 3 emissions are all indirect emissions (both upstream and downstream emissions not included in scope 2) that occur in the value chain of the reporting company. Examples are emissions associated with the production and transportation of purchased products or the use of sold products.

The description of a company’s organizational boundary (mostly through operational control, financial control and equity share) is relevant in determining the activities that are within its boundary, hence the classification of its direct and indirect emissions in the company’s value chain. The concept of scope makes calculating activities easier; benchmarking and comparison among organizations as emissions arising from different activities can be classified [31].

Methods of quantifying indirect scope 2 and 3 emissions have little consistency in policymaking. In the IPCC’s fifth assessment report [32], they emphasized the importance of scope 3 emissions with an assessment method using consumption-based carbon accounting based on EEMRIOs [33]. For organizations and industrial clusters, however, the use of EEMRIOs for scope 3 emissions is no easy task considering the lack of spatial data in the IO tables and more accessible methods are required.

## 3. Challenges of carbon accounting

Abating emissions in industrial supply chains is necessary to keep global warming to less than 1.5°C; achieving this will require adequate data through standardizing carbon accounting across the various protocols, platforms and standards in operation today [34]. We find a number of challenges within the current carbon accounting landscape, which we have categorized here.

### (a) Too many standards, methods and tools

The plethora of carbon accounting standards, methods and tools that are neither interoperable nor comparable is a challenge to the industry and could slow the transition to net zero. These standards/methods vary in terms of complexities and methodologies [1] and arise from different operational levels, such as sectoral, corporate and national, and from a mix of compliance and voluntary-based mechanisms [35]. Energy Systems Catapult [35] noted the lack of effective coordination needed to ensure emissions are comparable across accounting practices and traceable through supply chains because of the current overly complex carbon accounting ecosystem. Forty per cent of all GHG emissions are associated with industrial supply chains and are 5.5 times more than the direct emissions from the business’s own assets and operations [34]. Notwithstanding that standards such as the ISO, BSI and GHG protocol allow a significant level of flexibility in the carbon accounting methodologies and boundaries applied, emissions abatement stages are not always clear; hence, they limit the transparency, comparability and consistency of disclosed emissions [35]. Energy Systems Catapult further noted that even though there is a need for some sort of variation in accounting practices, there should be an overriding acceptable standard to improve the consistency of emissions disclosures. Kaplan & Ramanna [36] observed that the assumptions permitted to be made, and the general profligate use of secondary and industry averages data to calculate scope 3 emissions, do not allow for credible, accurate numbers, undermining its integrity.

Consider companies in the same sector using different accounting tools or even a company with complex supply chains using different accounting tools across different parts of the system; besides being more difficult both upstream and downstream, the accuracy of the emission is also questionable. The Rocky Mountain Institute [34] noted that with the different methods it is hard to produce meaningful and transparent results. Different rules characterize the methodologies for calculating emissions and determining contributions against targets, depending on which framework or standard is being reported against.

### (b) Multiple and non-interoperable data and data formats

Making emissions data interoperable, comparable, auditable and machine-readable via open standards is an absolute requirement to lower GHG emissions and secure a clean and sustainable ecosystem. Platforms like CDP and EcoVadis are very useful and present useful tools, but their data coverage is mostly organizational level and is annually timed. Currently, getting emission data is a yearly exercise, timed around the release of sustainability reporting because of the lack of accepted standards and format. Businesses use bespoke software, supplier data, central databases and manual spreadsheets to compile and track emissions data and obtain proxy emissions calculations from the GHG inventory [37,38]. However, site-level assessment may not adequately account for emissions of multiple product sites; more timely and geographically granular data (at entity and asset level) that can be used with assurance through verification and audit mechanisms is needed.

### (c) Lack of unified oversight

The UK environmental and carbon sectors have policy and advisory bodies like the Committee on Climate Change (CCC), which ensures that emissions targets are evidence-based and independently assessed; Department for Energy Security & Net Zero (DESNZ), which is responsible for ensuring secure energy and promoting action on climate change in the UK and internationally; and Department for Environment Food, and Rural Affairs (DEFRA), which develops the National Adaptation Programme that addresses the risks set out in the most recent UK Climate Change Risk Assessment [39]. DESNZ works with 14 agencies and public bodies, among which are CCC and Low Carbon Contracts Company; DEFRA works with 34 agencies, among which are Environment Agency and Office for Environment Protection [40]. The Environment Agency is the registry and oversees and enforces the Monitoring, Reporting and Verification (MRV) of policy mechanisms like the CCA, UK ETS, ESOS and ESCR. However, there is no central carbon regulator or group to oversee or to facilitate (i) economy-wide standardized MRV practices, (ii) a possible single disclosure point and (iii) to carry out oversight function of the different policy requirements to account for emissions like the California Air Resources Board economy-wide carbon regulation, oversight and supervision across all carbon policies within California.

### (d) Overburdening administrative procedures

Industries and businesses seem to be under severe administrative pull, consequent upon multiple standards and disclosure mechanisms (formal and informal). These standards with varying methodology and flexibilities & disclosure mechanisms with varying requirements, metrics and MRV processes engender complexities, reduce transparency and create extra administrative burden and cost. Pulls in different direction means extra costs to companies [2]. In their report on carbon accounting in the industry [35], Energy Systems Catapult noted that practices of standardization over time increased the administrative burden on industries as they were developed in isolation and without consideration of the whole system of emissions disclosures that industries are already engaged in. It also noted that existing verification processes are not well aligned and streamlined across the various mechanisms that industries are required to report to. Having verifiable empirical data can help manage reputational risk and enable investor confidence; however, organizations often indicate that engaging with verification processes, bodies and standards is costly, administratively challenging and time-consuming. It observed that the focus should be to reduce points of disclosure, overlap and administrative burden from different disclosure mechanisms and metrics. Aligning these frameworks so that they work together within a national context vis-à-vis an international context constitutes the real challenge.

### (e) Limitations of scope 3

Current global standards now include guidance for the incorporation of scope 3 categories on corporate carbon reporting, but it is up to the company to determine which categories it includes, which may be dependent on the availability of data or the relevance of the categories to the company’s business model [31,37]. To estimate scope 3 emissions, current GHG protocol methods require companies to estimate the scope 1 emissions of all their direct and indirect suppliers and customers. Besides the problem of multiple counting, the feasibility of the process, especially for companies with complex supply chains and national and global distribution networks, is in doubt [36]. These processes are cumbersome and complex, especially for small- and medium-scale businesses. The verification of scope 3 emissions, if data exist in several voluntary mechanisms, is quite challenging; hence, it is unreliable or based on average industry data [7,37]. The use of average industry and secondary data results in corporate greenwashing by competitors who also take credit for GHG reduction without changing their product design, procurement processes and energy consumption [36]. There are reservations about the validity of scope 3 reporting; this is evidenced by the fact that no ETS system trades on scope 3 emissions. Even the US Security and Exchange Commission (SEC) provided a litigation haven for businesses that voluntarily provide scope 3 disclosures. SEC have this to say, ‘The proposed rules would provide a safe harbour for liability from Scope 3 emissions disclosure and an exemption from the Scope 3 emissions disclosure requirement for smaller reporting companies’ [41].

Besides, Kaplan & Ramanna [36] and Energy Systems Catapult [37] noted that current major carbon accounting standards lack the ability to account more accurately for scope 3 emissions in the value chain and supply chain operations of businesses/industries, adducing that they fell short in their scope 3 standard and therefore discouraged supply-chain decarbonization.

Double counting or double claiming occurs when ownership of a single GHG reduction within the same scope is claimed by two or more companies. Classification of emissions as scope 1, scope 2 and scope 3 saves companies from double counting within scope 1 and scope 2. Scope 1 of one company could be scope 2 or 3 of another company. For instance, scope 1 emissions of a power producer are scope 2 emissions of an appliance user who uses that power, which are in turn the scope 3 emissions of both the appliance manufacturer and the appliance retailer.

Some levels of double counting are intrinsic in scope 3 accounting, and companies may accept double counting within scope 3 for the purposes of reporting scope 3 emissions to stakeholders, facilitating reductions in value chain emissions and tracking progress towards a scope 3 reduction target. Double counting within scope 3 occurs when emissions from a single emissions source are accounted for by two or more entities in the same value chain, sometimes these emissions are accounted for in different scope 3 categories; for example, a manufacturer and a retailer may both account for the scope 3 emissions from transportation of goods between them by a third party. Scope 3 accounting assists the mutual action of multiple entities to reduce emissions; however, to ensure transparency, companies should acknowledge potential double counting to ensure the accuracy of data and prevent double crediting by contractual agreement asserting sole ownership of reduction. Authorities should also avoid scope 3 emissions aggregation across companies to determine total emissions in each region [31].

## 4. Net-zero policy bedrock

The UK approach and modality to tackling climate change is underpinned by its Climate Change Act 2008 (2050 Target Amendment) Order 2019 [42]. The Climate Change Act commits to significantly reducing carbon dioxide and other GHG emissions to 100% of the 1990 level (net-zero target) [39]. Determined to achieve all the milestones (carbon budget) in the transition to net zero, the government updated the net-zero strategy: Build Back Greener [43] in March 2023 with Power Up Britain: Government’s blueprint for energy in the UK [40], by bringing together the UK Energy Security Plan and Net-Zero Growth Plan. The UK Energy Security Plan details how the UK will decarbonize and domesticate energy production and security by investing in renewables and nuclear, and the Net-Zero Growth Plan outlines how the UK will take advantage of the inherent opportunity, to diversify and decarbonize [40]. Other statutory documents that underpinned the UK effort towards net zero and mainstreamed into net-zero strategy are as follows: Build Back Greener and Power Up Britain include but not limited to Environmental Act (2021), Carbon Budget Deliveries Plan (March 2023), Energy Act (October 2023), Green Finance Strategy (March 2023), British Energy Security Strategy (April 2022), Transport Decarbonisation Plan (July 2021), Industrial Decarbonisation Strategy (March 2021), Hydrogen Strategy (August 2021) and Heat and Building Strategy (October 2021) (House of Commons, 2023).

The UK government observed that the path to net zero in the UK, outlined in the net-zero strategy, is still the right one. Beside other details, it essentially targets the following: (i) Power: powering the UK entirely with clean energy by 2035 subject to energy security; (ii) Fuel Supply and Hydrogen: deliver 5 GW of hydrogen production capacity by 2030 and minimize GHG emission in the oil and gas sector through the revised Oil and Gas Authority strategies; (iii) Industry: capture about 20–30 MtCO_2_ (million tonnes of carbon dioxide) across the economy, including 6 MtCO_2_ of industrial emissions, per year by 2030 by delivering four CCUS clusters; (iv) Heat and Buildings: set a path to all new heating appliances in homes and workplaces from 2035 being low carbon; (v) Transport: remove all road emissions at the tailpipe and kickstart zero emissions international travel; (vi) Natural Resources, Waste and Fluorinated Gases: support low-carbon farming and agricultural innovation and triple the woodland creation in line with the 2037 pathway; (vii) Greenhouse Gas Removals: balance residual emissions from hardest to decarbonize sector by deploying at least 5 MtCO_2_/yr of engineered Greenhouse Gas Reduction Technologies by 2030; and (viii) Cross-cutting Action: support the transition with a cross-cutting action to facilitate private finance through the UK infrastructure bank, annual progress update against a set of key indicators and the introduction of a new sustainability disclosures regime, including mandatory climate-related financial disclosures and a UK green taxonomy [40]. Currently, keeping to its commitment to become the world’s first net zero-aligned financial centre, the UK government has promised to (i) consult on the extension of transition planning disclosure requirements to the largest private companies, complementing existing requirements put in place by the Financial Control Authority (FCA); (ii) issue a call for evidence on scope 3 emissions reporting; and (iii) support the work of the International Sustainability Standards Board by setting a framework to address their suitability for adoption in the UK as soon as final standards are published [40].

Table 2 describes decarbonization drivers, characterized into organization/instrument of operation, coverage in terms of jurisdiction, objectives and perception in literature.

**Table 2.** Decarbonization drivers, characterized into organization/instrument of operation, coverage in terms of jurisdiction, objectives and perception in literature.

decarbonization drivers	organizations/ instruments	jurisdiction	objectives/perception
standards	GHG protocol	global/regional	standards setting. Fell short in scope 3 standards, discourages supply-chain decarbonization
	ISO (BSI)	global, sectoral, national (UK)	
disclosure mechanisms	CDP	global, regional, sectoral	runs the global disclosure system for investors, companies, cities, states and regions to measure and manage their environmental impacts. Data are organizational level and are annually timed
	ISSB (TCFD, CDSB, SASB) FSB—IFRS	global, work with regional and national body	the UK supports ISSB and plans to address their suitability for adoption
	SBTi	global	provide a clearly defined pathway (science-based) for companies to reduce greenhouse gas (GHG) emissions. Does not currently assess targets for cities, local governments, public sector institutions, educational institutions or non-profit organizations
	SECR, ESOS and CCA	UK	policy disclosure mechanism. Varying disclosure requirements and metrics
carbon pricing mechanism	compliance market-based mechanism—ETS (EU/UK/Québec ETS)	regional, national, city-wide	government sets the cap, determines the sectors to cover and distributes tradable allowances free or by auction. Targeted emissions reductions can be met cost-effectively; however, governmental regulation interferes with business and cost dynamics
	voluntary market-based mechanism (CORSIA, air carbon exchange (ACX) and UN carbon offset platform)	global, sectoral	carbon credits are traded voluntarily and represent reductions or removals of greenhouse gas emissions taking place outside of a company’s operation. It has great potential for greenwashing
	carbon tax	national, city-wide	government sets a tax rate and entities covered by the tax must pay this amount for every tonne they emit. Targeted emission reduction outcome is not guaranteed

### (a) Market-based mechanisms

The Kyoto Protocol of 1997 facilitated market-based mechanisms to support signatories to reduce their greenhouse gas (GHG) emissions. This includes the Clean Development Mechanism, where countries who have targets might ‘offset’ emissions in developing countries; Joint Implementation Mechanisms: both countries must have a target to jointly invest in carbon reduction projects; and Emissions Trading Scheme (ETS), also known as ‘Cap-and-Trade’ [44]. In an ETS, the regulatory body sets a ‘cap’: an upper limit of emissions for all participating industries and reduces it over time until total emissions fall. There is an option to offset polluting activities by buying additional allowance/carbon credit from participating companies/installations. Currently, there are 48 ETSs worldwide, 29 are in force, 11 are under consideration and eight are under development. The ETS is preferable to other carbon pricing instruments such as carbon tax because it assures the mitigation outcome of the policy by putting in place a ‘cap’: the total amount of emission allowable in the sector [45].

Table 3 is a comparison of the UK ETS, EU ETS and China ETS using common parameters.

**Table 3.** Comparison between UK ETS, EU ETS and China ETS.

parameters	decarbonization driver (ETS)		
**location**	UK	EU	China
start of operation	2021	2005	2021
sector coverage	domestic aviation industry, power	domestic aviation industry, power	power
cap	147.2 MtCO_2_e (2023)	1529 MtCO_2_e (2022, stationary installations), 28.4 MtCO_2_e (2022, aviation)	~4500 MtCO_2_e (2019 and 2020 each)
GHG reduction target	by 2030: at least a 68% reduction in UK net GHG emissions from 1990 levels, including emissions from LULUCF (UK NDC, December 2020) by 2050: net-zero UK GHG emissions	by 2030: at least 55% below 1990 GHG levels by 2050: climate neutrality	by 2030: peak CO_2_ emissions. Before 2030: lower CO_2_ emissions per unit of GDP by over 65% from 2005 levels by 2060: carbon neutrality
coverage (% GHG emissions covered)	26	38	44%
allowance price (USD per MtCO_2_e)	93	83	8
auction share (% of 2022 cap)	54	57	0
offset use (0–20%)	0	0	5
GHGs covered	CO_2_, N_2_O, PFCs	CO_2_, N_2_O, PFCs	CO_2_
point of regulation	point source	point source	point source (power); downstream (indirect emissions from electricity and heat consumption)
number of entities	1006 entities (2021)	number of entities 8757 stationary installations 371 aircraft operators	2162 (2020 and 2021)
overall GHG emissions excl. LULUCF (MtCO_2_e)	405.8	3293.1	12 301 (2014)
verified ETS emissions	102.60 MtCO_2_e	1335.00 MtCO_2_e	

Source: Data from ICAP [45].

MtCO_2_e, million tonnes carbon dioxide equivalent.

The UK ETS mirrors the EU ETS in operationality, compliance and MRV. The EU ETS is older, bigger, the first and best known; however, the details show that the UK is a jurisdiction with stricter climate regulations and more ambitious emissions targets. Besides the above, the point of regulation is at the installation level (point source) indicating that they are mostly scope 1 and 2. The UK ETS authority has the overall responsibility for designing and implementing the UK ETS. It is composed of the representatives of the UK Government, Scottish Government, Welsh Government and the Department of Agriculture, Environment and Rural Affairs of Northern Ireland. The Environment Agency serves as the registry administrator responsible for the management of accounts in the UK Emissions Trading Registry and the national regulator responsible for enforcing compliance with the UK ETS regulations [45].

### (b) Disclosure mechanisms

Disclosure-based mechanisms encourage decarbonization by increasing the visibility of operators’ polluting activities through measurement, reporting and verification. They can be compliant based in response to regulatory, contractual and corporate responsibilities or voluntary [37]. Table 4 shows some disclosure mechanisms and their characteristics.

**Table 4.** Carbon disclosure mechanisms.

disclosure-based mechanism/ regulatory authority/ jurisdiction	objective	disclosure requirement	MRV guidance
CDP Global	disclose investors, companies, cities, regions and states’ environmental impact and assist in measuring and managing risks and opportunities on climate change, water security, deforestation and biodiversity	client to report emissions in CO_2_e (carbon dioxide equivalent) or by other metrics (e.g. revenue) and other environmental impact via an online system	verification through an accredited third-party external organization according to verification standard
ISSB (TCFD, CDSB, SASB) IFRS global and intend to work with jurisdiction and national regulator	disclose sustainability-related (climate-related) financial information that presents risks and opportunities in the short, medium and long-term investors	disclose governance, risk management, strategy, metrics and target used by the organization to assess climate-related risks and opportunities in line with its strategy and risk management process. Disclose scope 1, scope 2 and, if appropriate, scope 3 greenhouse gas emissions and the related risks	proportionate and enforceable. The UK FCA has aligned with TCFD. The UK working to access/adopt ISSB disclosure standard in the UK financial sector requirement
SECR UK Environment Agency	SECR policy requires the disclosure of energy use, energy efficiency steps and carbon emissions information in organizations’ annual reports. It follows guidelines set out in the GHG Protocol and supports the use of UK Government GHG Inventory conversion factors	varies for quoted and large companies. Energy efficiency steps and the methodology are to be disclosed by quoted and mid-large companies. Quoted companies are expected to also disclose global energy use and derived emissions. Scope 1, 2 and 3 reporting requirements are dependent on company size and energy use	methodology must be disclosed, but do not need to be independently verified. It includes sector-specific guidance, documentation and flexibility to suit different business models
ESOS/ UK/ Environmental Agency	ESOS is a policy designed to ensure that large UK companies are energy efficient. It focuses on companies with more than 250 employees, or a turnover greater than €50 m and a balance sheet greater than €43 m	Required to show the energy supplied and consumed (kWh or spend £) through mandatory energy audits	reports are verified through mandatory energy audits every 4 years by an ESOS assessor and consist of annually 12 consecutive monthly data
CCA/ UK/ Environmental Agency	the CCA is a voluntary scheme, an agreement between UK industry sector associations and the Environment Agency to reduce emissions for specified industrial processes in return for a reduction in the Climate Change Levy (CCL—a consumption tax levied on energy bills)	companies are required to report energy use and carbon emissions from eligible processes, especially energy-intensive processes in manufacturing	audits are carried out each year on selected operators to verify eligibility and performance. Annual reporting is expected to meet an energy saving target measured at every 2-year target period

### (c) Carbon accounting standards

Standards are guidelines, specifications, requirements, assumptions and methods defined by a body or organization that can be used consistently to ensure that processes, services, materials and products are fit for purpose. A standard could be statutory or generally consented to. They provide a basis to measure and evaluate performance and promote interoperability and comparability [35].

Major GHG emissions accounting standards are GHG protocol and ISO (BSI). These organizations are already providing some level of standardized basis for corporate carbon accounting and reporting and are currently being implemented by some countries and businesses [37]. However, these standards, especially the GHG protocol, the de facto global greenhouse gas emission accounting standard, fell short in its scope 3 and therefore discourage supply-chain decarbonization [36].

## 5. Framework considerations

**Figure 1.**
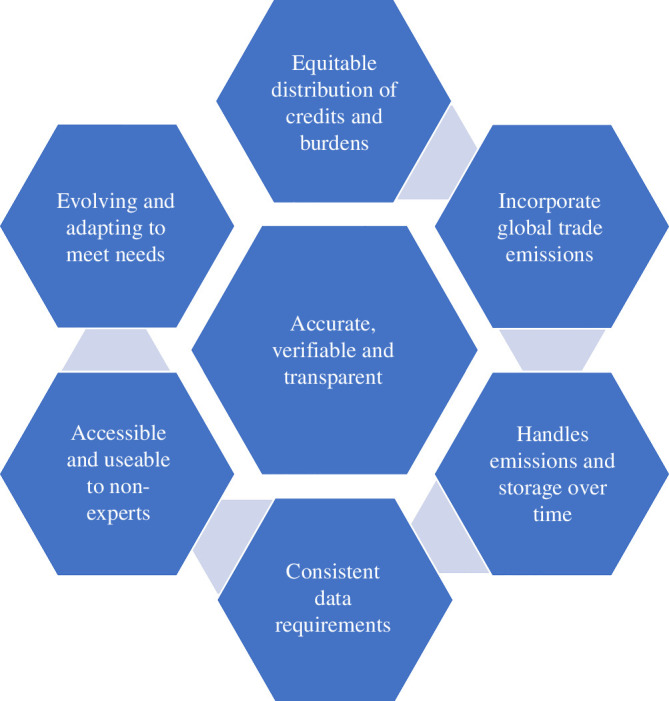
Proposed requirements for a carbon framework.

Any carbon accounting method and activity should prioritize global emission reduction and the creation of a just transition rather than compensation to any one company or country. The particular method should be selected by considering the principles of environmental justice and the obligation to compensate for any damage, the environmental impact generated or benefits gained from emissions as a result of the economic activity [4]. However, carbon accounting approaches are often selected based on industry or sector norms, or regulatory or policy requirements, with associated methods embedded (figure 1).

Businesses, industry and supply chains do not work independently; therefore, a system that achieves the overarching aim while rewarding (or penalizing) the appropriate actors is critical. Therefore, a robust framework is required.

We propose that the framework consists of seven key components, with the requirement for the approach to be accurate, verifiable and transparent at its heart. Round the outside are the key aspects required to ensure that a framework is equitable and can evolve as needed. Critical to success is reducing the burden on industry and business; this should not require gathering more data, simply using the existing data more efficiently and consistently. Furthermore, as CCUS becomes more commonplace, any framework must be able to account for the carbon stored over variable time frames and determine the associated burdens and credits. Similarly, a framework needs to be able to incentivize companies to work together where appropriate and be robust enough to meet the need of decarbonizing the complex nature of industries, businesses and clusters.

In order to make data available and accessible to all users along the supply chain and to meet the different requirements of different stakeholders without adding a significant burden to businesses and industries, a distributed digital system’s architecture could be deployed. This would assist in accounting for emissions across the life cycle of a product while supporting the MRV requirements of specific policy mechanisms and ensuring the protection of commercially sensitive data for industries [35].

Core to success is the ability to be flexible while also dealing with the level of uncertainty inherent in data across sectors and countries.

## 6. Conclusions

Climate change is one of the most pressing issues facing humanity. Meeting the net-zero targets set in response to this emergency requires a robust and consistent approach. Therefore, counting and accounting for carbon is important, and yet is shown to be complex and varied in approach. This results in the possibility of double counting, avoiding counting and making decisions based on localized credits, which result in higher global emissions. Clearly, this is not the desired outcome.

With the analysis of the shortcomings of the existing approaches, this paper highlights and identifies the accounting methods used across the key carbon accounting standards, regulations and policies used across multiple sectors. This shows the deficit of consistency of approaches and highlights why the different methods can result in confusion, complexity and the prioritization of behaviour to meet legislation or policy that might not actually reduce global carbon emissions. Critical issues for a carbon framework are identified.

The selection of the carbon accounting method should prioritize the urge to fight climate change and redistribution of justice rather than compensation for individual actors. To do this, the needs of the individual need to be identified so that appropriate credits and burdens can be allocated, incentivizing the required carbon emission reduction.

## Data Availability

This article has no additional data.

## References

[1] Plassmann K , Norton A , Attarzadeh N , Jensen MP , Brenton P , Edwards-Jones G . 2010 Methodological complexities of product carbon footprinting: a sensitivity analysis of key variables in a developing country context. Environ. Sci. Policy **13** , 393–404. (10.1016/j.envsci.2010.03.013)

[2] Dixit MK , Fernández-Solís JL , Lavy S , Culp CH . 2012 Need for an embodied energy measurement protocol for buildings: a review paper. Renew. Sustain. Energy Rev. **16** , 3730–3743. (10.1016/j.rser.2012.03.021)

[3] Matthews HD , Zickfeld K , Koch A , Luers A . 2023 Accounting for the climate benefit of temporary carbon storage in nature. Nat. Commun. **14** , 5485. (10.1038/s41467-023-41242-5) 37679349 PMC10485027

[4] Steininger KW , Lininger C , Meyer LH , Muñoz P , Schinko T . 2016 Multiple carbon accounting to support just and effective climate policies. Nat. Clim. Chang **6** , 35–41. (10.1038/nclimate2867)

[5] Li Y , Yang X , Du E , Liu Y , Zhang S , Yang C , Zhang N , Liu C . 2024 A review on carbon emission accounting approaches for the electricity power industry. Appl. Energy **359** , 122681. (10.1016/j.apenergy.2024.122681)

[6] Samuel G , Lucivero F , Knowles B , Wright K . 2024 Carbon accounting in the digital industry: the need to move towards decision making in uncertainty. Sustainability **16** , 2017. (10.3390/su16052017) 39286603 PMC7616451

[7] Catapult ES . 2022 Developing the UK emissions trading scheme. Birmingham, UK: Energy Systems Catapult.

[8] Karakaya E , Yılmaz B , Alataş S . 2019 How production-based and consumption-based emissions accounting systems change climate policy analysis: the case of CO_2_ convergence. Environ. Sci. Pollut. Res. **26** , 16682–16694. (10.1007/s11356-019-05007-2) 30989611

[9] Tukker A , Pollitt H , Henkemans M . 2020 Consumption-based carbon accounting: sense and sensibility. Clim. Pol. **20** , S1–S13. (10.1080/14693062.2020.1728208)

[10] Afionis S , Sakai M , Scott K , Barrett J , Gouldson A . 2017 Consumption‐based carbon accounting: does it have a future? WIREs. Clim. Change **8** . (10.1002/wcc.438)

[11] de Boer BF , Rodrigues JFD , Tukker A . 2019 Modeling reductions in the environmental footprints embodied in European Union’s imports through source shifting. Ecol. Econ. **164** , 106300. (10.1016/j.ecolecon.2019.04.012)

[12] Wang Z , Li Y , Cai H , Wang B . 2018 Comparative analysis of regional carbon emissions accounting methods in China: production-based versus consumption-based principles. J. Clean. Prod. **194** , 12–22. (10.1016/j.jclepro.2018.05.018)

[13] Zhu C , Guo G , Su S , Hong J , Li X . 2023 Multiple accounting of carbon emission responsibility in the construction sector under different principles: a study from China. Renew. Sustain. Energy Rev. **186** , 113651. (10.1016/j.rser.2023.113651)

[14] IEEP, TRINOMICS, IVM, UNEP-WCMC . 2021 Methodology for assessing the impacts of trade agreements on biodiversity and ecosystems. Brussels, Belgium/London, UK: Institute for European Policy.

[15] Davis SJ , Caldeira K . 2010 Consumption-based accounting of CO_2_ emissions. Proc. Natl Acad. Sci. USA **107** , 5687–5692. (10.1073/pnas.0906974107) 20212122 PMC2851800

[16] Zheng S , Yang J , Chen C , Wu B . 2023 Embodied carbon accounting for forest industry trade in BRICS countries: an MRIO modeling approach. Sustainability **15** , 12503. (10.3390/su151612503)

[17] Zhongxiu Z , Yunfeng Y . 2014 Consumption-based carbon emissions and international carbon leakage: an analysis based on the WIOD database. Soc. Sci. China **35** , 174–186. (10.1080/02529203.2014.927111)

[18] Liang S , Qu S , Zhu Z , Guan D , Xu M . 2017 Income-based greenhouse gas emissions of nations. Environ. Sci. Technol. **51** , 346–355. (10.1021/acs.est.6b02510) 27936320

[19] Leontief WW . 2016 Input-output economics. London, UK: Palgrave Macmillan. (10.1057/978-1-349-95121-5_1072-1)

[20] Miller RE , Blair PD . 2009 Input-output analysis: foundations and extensions. Cambridge, UK: Cambridge University Press. (10.1017/CBO9780511626982)

[21] Acquaye A , Feng K , Oppon E , Salhi S , Ibn-Mohammed T , Genovese A , Hubacek K . 2017 Measuring the environmental sustainability performance of global supply chains: a multi-regional input-output analysis for carbon, sulphur oxide and water footprints. J. Environ. Manage. **187** , 571–585. (10.1016/j.jenvman.2016.10.059) 27876164

[22] Barrett J , Peters G , Wiedmann T , Scott K , Lenzen M , Roelich K , Le Quéré C . 2013 Consumption-based GHG emission accounting: a UK case study. Clim. Pol. **13** , 451–470. (10.1080/14693062.2013.788858)

[23] Wiedmann T , Lenzen M . 2018 Environmental and social footprints of international trade. Nat. Geosci. **11** , 314–321. (10.1038/s41561-018-0113-9)

[24] Dorninger C , Hornborg A . 2015 Can EEMRIO analyses establish the occurrence of ecologically unequal exchange? Ecol. Econ. **119** , 414–418. (10.1016/j.ecolecon.2015.08.009)

[25] Bellora C , Fontagné L . 2023 EU in search of a carbon border adjustment mechanism. Energy Econ. **123** , 106673. (10.1016/j.eneco.2023.106673)

[26] European Commission . 2023 Carbon Border Adjustment Mechanism. Brussels, Belgium: European Commission. See https://taxation-customs.ec.europa.eu/carbon-border-adjustment-mechanism_en.

[27] ISO 14040. BS EN ISO 14040 . 2006 Environmental management - life cycle assessment - principles and framework. London, UK: International Standards Organisation.

[28] ISO 14044. BS EN ISO 14044:2006 . 2006 Enivronmental management - life cycle assessment - requirements and guidelines. London, UK: International Standards Organisation.

[29] ISO 14025. BS EN ISO 14025 . 2010 Environmental labels and declarations type III environmental declarations principles and procedures. London, UK: International Standards Organisation.

[30] EN 15804. BS EN 15804 . 2019 Sustainability of construction works environmental product declarations core rules for the product categories of construction works. London, UK: British Standards Institute.

[31] GHG Protocol . 2014 Policy and action standard: an accounting and reporting standard for estimating the greenhouse gas effects of policies and actions. Available from: https://ghgprotocol.org/sites/default/files/standards/Policy%20and%20Action%20Standard.pdf

[32] IPCC . 2015 Climate change 2014: synthesis report. Geneva, Switzerland: The Intergovernmental Panel on Climate Change.

[33] Hertwich EG , Wood R . 2018 The growing importance of scope 3 greenhouse gas emissions from industry. Environ. Res. Lett. **13** , 104013. (10.1088/1748-9326/aae19a)

[34] RMI . 2020 The next frontier of carbon accounting. Basalt, CO: Rocky Mountain Institute.

[35] ES Catapult . 2023 Carbon accounting and standards in industry: a framework for innovation and growth. UK: Energy Systems Catapult.

[36] Kaplan RS , Ramanna K . 2021 Accounting for climate change. Harvard Business Review. See: https://hbr.org/2021/11/accounting-for-climate-change.

[37] ES Catapult . 2022 Carbon accounting in industry: learning from the South Wales industrial cluster to develop a consistent and coherent national framework. UK: Energy Systems Catapult.

[38] BEIS . 2022 Towards a market for low emissions industrial products: call for evidence summary of responses. London, UK: Department for Business, Energy & Industrial Strategy.

[39] CCC . 2019 Net zero: the UK’s contribution to stopping global warming. London, UK: Committee on Climate Change.

[40] HM Government . 2023 Powering up Britain: the net zero growth plan. London, UK: HM Government.

[41] U. S. Securities and Exchange Commission . 2022 SEC proposes rules to enhance and standardize climate-related disclosures for investors. Press release, Washington DC.

[42] The Climate Change Act 2008 (2050 target amendment) order 2019. See: https://www.legislation.gov.uk/uksi/2019/1056/article/1/made

[43] HM Government . 2021 Net zero strategy: build back better. London, UK: HM Government.

[44] He P , Dou G , Zhang W . 2017 Optimal production planning and cap setting under cap-and-trade regulation. J. Oper. Res. Soc. **68** , 1094–1105. (10.1057/s41274-016-0123-1)

[45] Compare ETS . 2023 International carbon action partnership. See https://icapcarbonaction.com/en/compare/99/55/43.

